# Advantage of endoscopic papillectomy for ampullary tumors as an alternative treatment for pancreatoduodenectomy

**DOI:** 10.1038/s41598-022-19439-3

**Published:** 2022-09-07

**Authors:** Shohei Abe, Arata Sakai, Atsuhiro Masuda, Mika Miki, Yoshiyuki Harada, Kae Nagao, Noriko Inomata, Shinya Kohashi, Hisahiro Uemura, Shigeto Masuda, Shigeto Ashina, Masanori Gonda, Kohei Yamakawa, Masahiro Tsujimae, Yasutaka Yamada, Takeshi Tanaka, Takashi Kobayashi, Ryota Nakano, Hideyuki Shiomi, Daisuke Tsugawa, Hiroaki Yanagimoto, Tetsuo Ajiki, Maki Kanzawa, Takumi Fukumoto, Tomoo Itoh, Yuzo Kodama

**Affiliations:** 1 Division of Gastroenterology, Department of Internal Medicine, Kobe University Graduate School of Medicine, 7-5-2 Kusunoki-cho, Chuo-ku, Kobe, Hyogo 650-0017 Japan; 2 Division of Gastroenterology and Hepatobiliary and Pancreatic Diseases, Department of Internal Medicine, Hyogo Medical University, 1-1 Mukogawa-cho, Nishinomiya, Hyogo 663-8501 Japan; 3 Division of Hepato-Biliary-Pancreatic Surgery, Department of Surgery, Kobe University Graduate School of Medicine, 7-5-2 Kusunoki-cho, Chuo-ku, Kobe, Hyogo 650-0017 Japan; 4 Division of Diagnostic Pathology, Kobe University Graduate School of Medicine, 7-5-2 Kusunoki-cho, Chuo-ku, Kobe, Hyogo 650-0017 Japan; 5 Division of Gastroenterology, Department of Internal Medicine, Kobe University Graduate School of Medicine, 7-5-2 Kusunoki-cho, Chuo-ku, Kobe, 650-0017 Japan

**Keywords:** Gastroenterology, Oncology

## Abstract

Endoscopic papillectomy for early ampullary tumors is considered a minimally invasive and useful alternative to pancreatoduodenectomy; however, its indications remain unclear. This study aimed to clarify the advantages of endoscopic papillectomy by investigating the clinical outcomes of patients who underwent endoscopic papillectomy or pancreatoduodenectomy for early ampullary tumors. Patients diagnosed with early ampullary tumors (adenoma, Tis, T1a) who underwent endoscopic papillectomy or pancreatoduodenectomy between June 2008 and October 2019 were included, and their clinical outcomes were analyzed. Seventy-four patients (34 patients with adenomas and 40 patients with adenocarcinomas) were divided into two groups, namely endoscopic papillectomy (n = 43) and pancreatoduodenectomy (n = 31). The estimated 5-year overall survival rate of all early ampullary tumors was 92%. Complete resection rate was significantly lower for endoscopic papillectomy patients versus pancreatoduodenectomy patients (48.8% vs. 100%; p < 0.001). Recurrence was more common in the endoscopic papillectomy group compared to the pancreatoduodenectomy group (16.3% vs. 3.2%; p = 0.128), but all recurrences were controllable by endoscopic treatment. The median length of hospital stay for the endoscopic papillectomy group was significantly shorter compared to the endoscopic papillectomy group (11 days vs. 42 days; p < 0.001). The Comprehensive Complication Index was significantly lower in the endoscopic papillectomy group compared to the pancreatoduodenectomy group (14.8 vs 22.6%; p = 0.002). Endoscopic papillectomy for early ampullary tumors is useful and may be an alternative treatment for pancreatoduodenectomy in selected cases.

## Introduction

Ampullary neoplasms are rare, accounting for less than 0.5% of all gastrointestinal neoplasms^1^. Histologic analysis revealed that more than 90% of ampullary neoplasms are adenomas and adenocarcinomas^2^. As ampullary adenomas follow an adenoma-carcinoma sequence, they have the potential for malignant transformation^3,4^. Because of their malignant potential, the indication for resection of ampullary neoplasms, regardless of their grade, is widely accepted.

Currently, the standard treatment for ampullary carcinoma is pancreatoduodenectomy (PD). The overall 5-year survival rate for patients with ampullary carcinoma below T1a cancer (limited to the sphincter of Oddi) who underwent PD has been reported to be 83–98%^5,6^. However, PD is still associated with a high mortality rate despite a recent reduction in the mortality rate to less than 5%^7,8^. In contrast, endoscopic papillectomy (EP) is considered a safe and minimally invasive alternative to surgery for the treatment of ampullary adenoma^9^. The prognosis of patients who received EP for ampullary adenoma has been reported to be favorable, with a mortality rate of 0–1%^10^. Europian Society Gastorointestinal Endoscopy (ESGE) recommends EP in patients with ampullary adenoma without intraductal extension^11^. Recently, EP may be useful and reliable for the curative treatment of ampullary carcinoma, in which the tumor is limited to mucosa of the ampulla of Vater^12^. However, 10% of lymph node metastases have been reported for T1a ampullary carcinoma^13^, and there is no consensus on performing EP for early stage ampullary carcinoma^11^. In addition, there has been no report of a direct comparison of the invasiveness of EP and PD for ampullary tumors.

We defined ampullary carcinoma below T1a, including adenoma, as “early ampullary tumors”. The aim of this study was to clarify the advantages of EP by investigating the clinical outcomes of patients who underwent EP or PD for early ampullary tumors. In addition, we believe that this study will contribute to the development of EP as an alternative treatment for ampullary tumors.

## Materials and methods

### Study design

This retrospective observational study included patients who underwent EP or PD for ampullary tumors at the Kobe University Hospital between June 2008 and October 2018. This study retrospectively investigated cases of early ampullary tumors (adenoma, Tis, T1a) with a final pathological diagnosis after the procedure. Patients with a follow-up period of less than 6 months were excluded. Patients who underwent additional PD after EP were also excluded because they received both treatments being compared in the study, which would have complicated the evaluation.

The study protocol was reviewed and approved by the ethics committee of the Kobe University Hospital (No.200160). The requirement of informed consent was waived because of the retrospective study design and the study information was disclosed on our hospital website, providing the eligible patients with an opportunity to opt out. This was also approved by the ethics committee of the Kobe University Hospital. This study was conducted in accordance with the Declaration of Helsinki. All authors had access to the study data, and reviewed and approved the final manuscript.

### Pre-procedural evaluation

Tumor staging and intraductal involvement were evaluated using computed tomography (CT) and endoscopic ultrasound in all patients. Intraductal ultrasonography was attempted in cases in which intraductal extension diagnosis by endoscopic ultrasound was difficult. Cases with adenoma diagnosed by a preoperative biopsy examination, without bile/pancreatic duct extension, were indicated for EP. PD was recommended for cases diagnosed as carcinoma by preoperative biopsy or adenomas with bile duct/pancreatic duct extension by endoscopic ultrasound/Intraductal ultrasonography; however, EP was performed for cases in which PD was not feasible due to poor general condition, old age, or comorbid disease when T2 invasion was not detected by preoperative imaging studies.

### Endoscopic and surgical procedures, pathological diagnosis, and follow up

EP was performed with a standard polypectomy snare using a blended electrosurgical current. Although piecemeal resection was performed, because of their size, the tumors were completely endoscopically resected in all cases. We attempted to insert both the bile duct and pancreatic duct stent after EP. Patients remained hospitalized until a duodenoscopy was performed 7 days after EP; if no bleeding or residual tumor was identified, the stent was removed. The first follow-up duodenoscopy after discharge was scheduled for three months after EP. Follow-up esophagogastroduodenoscopy/CT was scheduled every year for 5 years. If a recurrent lesion was diagnosed as adenoma on biopsy, endoscopic treatment, such as argon plasma coagulation (APC) or additional EP, was performed. PD was recommended for all cases of recurrent carcinoma, but patients who refused or were unfit for PD were treated endoscopically.

The surgical procedure consisted of PD with standard lymphadenectomy. Subtotal stomach-preserving pancreatoduodenectomy was performed in every PD cases. The reconstruction of the digestive tract was performed using a modified child method. After intervention, a CT scan was performed once a year.

All specimens, including endoscopic resections and surgical resections, were evaluated by experienced pathologists. The final depth of cancer invasion was recorded according to the classification of biliary tract carcinoma developed by the American Joint Committee on Cancer, 8th edition^14^ (Table 1).

**Table 1:** Definitions of ampullary tumor invasion by the American Joint Committee on Cancer, 8th edition.

Tx	Primary tumor cannot be assessed
T0	No evidence of primary tumor
Tis	Carcinoma in situ
T1a	Tumor limited to ampulla of Vater or sphincter of Oddi
T1b	Tumor invades beyond the sphincter of Oddi and/or into the duodenal submucosa
T2	Tumor invades into the muscularis propria of the duodenum
T3	Tumor directly invades into the pancreas (up to 0.5 cm) or extends into peripancreatic tissue
T4	Tumor involves the celiac axis, superior mesenteric artery or common hepatic artery

### Definitions

The primary outcome of this study was the estimated five-year overall survival (OS) of patients who underwent EP or PD for early ampullary tumors. Secondary outcomes included disease-specific survival (DSS), complete resection, recurrence, duration of hospital stay, readmission, and post-procedural complications.

Complete resection was defined as R0 resection, and in the case of EP, piecemeal resection was defined as R1 resection. Post-EP recurrence was defined as the presence of a new lesion on post-EP endoscopy and the diagnosis of a neoplastic lesion on endoscopic biopsy. Recurrence after PD was defined as a lesion found on a follow-up CT after surgery. The duration of hospital stay was calculated from the day of EP or PD until discharge. Readmission was defined as hospitalization for recurrence or adverse events.

Adverse events within 30 days after procedure were defined as early adverse events. Post-EP bleeding and pancreatitis were defined according to American Society Gastrointestinal Endoscopy (ASGE) guidelines^15^, and we used the revised Atlanta Classification for severity classification of pancreatitis^16^. Post-EP bleeding was defined as a decrease in hemoglobin level of > 3 g/dL associated with clinical evidence of bleeding. Bleeding during the procedure was not defined as post-EP bleeding. The definition of post-EP pancreatitis was that serum amylase levels to > 3 times normal at more than 24 h after the procedure, and that post-EP abdominal pain occurred. Perforation was defined on the basis of symptoms and abdominal CT.

Post-PD hemorrhage was defined and classified according to the International Study Group of Pancreatic Surgery^17^. Post-PD pancreatic fistula was defined and graded according to the criteria set by the International Study Group on Pancreatic Fistula^18^. Post-PD pancreatitis and bleeding defined grades B and C as positive. Furthermore, an analysis using the Clavien–Dindo Classification (CDC)^19^ was used as an index of common adverse events (Table 2). Severe adverse events were defined as those with a grade ≥ IIIb. CDC was used to calculate the Comprehensive Complication Index (CCI) using the online tool^20^.

**Table 2:** Clavien–Dindo classification.

Grade	Definition
Grade I	Any deviation from the normal postoperative course without the need for pharmacological treatment or surgical endoscopic, and radiological interventions Allowed therapeutic regimens are: drugs as antiemetics, antipyretics, analgetics, diuretics, electrolytes, and physiotherapy. This grade also includes wound infections opened at the bedside
Grade II	Requiring pharmacological treatment with drugs other than such allowed for grade I complications. Blood transfusions and total parenteral nutrition are also included
Grade III	Requiring surgical, endoscopic or radiological intervention
Grade IIIa	Intervention not under general anesthesia
Grade IIIb	Intervention under general anesthesia
Grade IV	Life-threatening complication (including CNS complications)^a^ requiring IC/ICU management
Grade IVa	Single organ dysfunction
Grade IVb	Multiorgan dysfunction
Grade V	Death of a patient
Suffix “d”	If the patient suffers from a complication at the time of discharge, the suffix “d”(for “disability”) is added to the respective grade of complication. This label indicates the need for a folloe-up to fully evaluate the complication

*CNS* central nervous system, *IC* intermediate care, *ICU* intensive care unit.

^a^Brain hemorrhage, ischemic stroke, subarrachnoidal bleeding, but excluding transient ischemic attacks.

Late adverse events were defined as ≥ 30 days after the procedure, requiring readmission for the treatment of the adverse events.

### Data analysis and statistics

All statistical analyses were conducted using JMP software (version 12, SAS Institute, Cary, NC, USA). Continuous variables are presented as median (range), depending on the data distribution. The χ^2^ test or Fisher’s exact test, when applicable, was used to compare frequencies. The Wilcoxon rank-sum test was used to compare skewed continuous variables. Survival data were recorded at the time of the final follow-up. The median survival of patients who underwent EP or PD for ampullary tumors was estimated using the Kaplan–Meier method. OS was defined as the time between treatment and death due to any cause. DSS was defined as the time between treatment and death due to ampullary tumor progression. All statistical tests were two-tailed, and statistical significance was set at P < 0.05.

### Ethics approval

The study protocol was reviewed and approved by the ethics committee of the Kobe University Hospital (No.200160). This study was conducted in accordance with the Declaration of Helsinki.

## Results

### Patients and baseline characteristics

**Figure 1:**
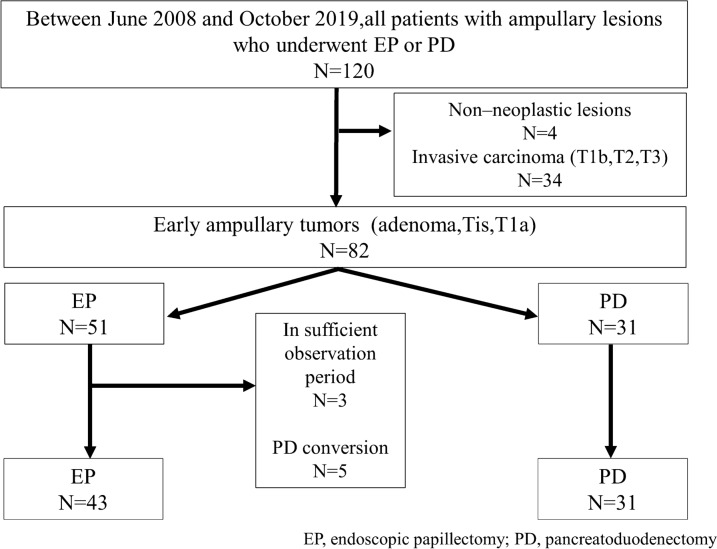
Study flowchart.

A total of 120 patients underwent EP or PD for ampullary tumors. Among them, 82 (51 EP and 31 PD) cases were diagnosed as early ampullary tumors in the final pathological diagnosis. In the EP group, 3 cases were excluded due to insufficient observation periods and 5 were excluded due to additional PD. Additional PD was required in four patients who were diagnosed with positive or uncertain resection margins of adenocarcinoma after EP and in one patient who was diagnosed with positive resection margins of adenoma after EP. Of the cases with additional PD, only one patient who was diagnosed with T1a ampullary carcinoma (limited to the sphincter of Oddi beyond the mucosa of the Ampulla of Vater) after EP had residual cancer after surgery. In the remaining four cases, no tumor was found in the resected specimens. There was no exclusion in the PD group. Finally, 74 cases, with 43 EP and 31 PD cases, were included in this study (Fig. 1). The baseline characteristics of the patients are shown in Table 3. In all categories, there was no significant difference between the EP and PD groups.

**Table 3:** The characteristics of all patients.

	ALL n = 74	EP n = 43	PD n = 31	p-value
Median age, years (range)	68 (37–88)	66 (37–88)	70 (41–81)	0.227
**Sex, n (%)**				
Male	51 (68.9)	29 (67.4)	22 (71.0)	0.803
Female	23 (31.0)	14 (32.6)	9 (29.0)	
History of drinking, n (%)	20 (27.0)	10 (23.3)	10 (32.3)	0.434
History of smoking, n (%)	29 (39.2)	17 (39.5)	12 (38.7)	1.000
Oral anticoagulans, n (%)	10 (13.5)	5 (11.6)	5 (16.1)	0.733
**Clinical presentation, n (%)**				
Incidental finding	50 (67.6)	33 (76.7)	17 (54.8)	0.077
Abdominal pain	22 (29.7)	10 (23.3)	12 (38.7)	0.199
Jaundice	2 (2.7)	0	2 (6.5)	0.172
**Associated diseases, n (%)**				
Hypertention	31 (41.9)	15 (34.9)	16 (51.6)	0.101
Diabetes mellitus	16 (21.6)	10 (23.3)	6 (19.4)	0.779
Cardiovascular disease	6 (8.1)	4 (9.3)	2 (6.5)	1.000
FAP	4 (5.4)	3 (7.0)	1 (3.2)	0.635

*FAP* familial adenomatous polyposis.

### Pathological findings

The pathological findings are shown in Table 4. The final pathological diagnoses for all patients were 34 (45.9%) adenomas and 40 (54.1%) adenocarcinomas (16 Tis, 24 T1a). In the EP group, there were 32 (74.4%) adenomas and 11 (25.6%) adenocarcinomas (8 Tis and 3 T1a). In the PD group, there were 2 (6.5%) adenomas and 29 (93.5%) adenocarcinomas (8 Tis, 21 T1a). Bile duct extension was detected by preoperative endoscopic ultrasound in both cases of adenoma that received PD. The number of adenomas was significantly higher in the EP group, and the number of adenocarcinomas was significantly higher in the PD group. Two patients in the PD group had lymphovascular invasion, both of which were T1a (limited to the sphincter of Oddi beyond the mucosa of the Ampulla of Vater). No lymph node metastases were observed. There was no significant difference in the histological type of adenocarcinoma between the groups. The median tumor size was 20 mm in both groups, but the difference was not significant.

**Table 4:** Pathological findings.

	ALL n = 74	EP n = 43	PD n = 31	p-value
**Type of tumors, n (%)**				
Adenoma	34 (45.9)	32 (74.4)	2 (6.5)	< 0.001
Adenocarcinoma	40 (54.1)	11 (25.6)	29 (93.5)	< 0.001
Tis	16 (40.0)	8 (72.7)	8 (27.6)	0.569
T1a	24 (60.0)	3 (27.3)	21 (72.4)	< 0.001
Lymphovasucular invasion	2 (5.0)	0	2 (6.9)	0.172
Lymph node metastasis	0	–	0	
**Histologic type of adenocarcinoma**				
Papillary adenocarcinoma	5 (12.5)	1 (9.1)	4 (13.8)	1.000
Well differentiated adenocarcinoma	33 (44.6)	10 (23.3)	23 (74.2)	0.649
Poorly differentiated adenocarcinoma	1 (2.5)	0	1 (3.4)	1.000
Mucinous adenocarcinoma	1 (2.5)	0	1 (3.4)	1.000
Median tumor size, mm (range)	20 (10–65)	20 (12–39)	20 (10–65)	0.779

*EP* endoscopic papillectomy, *PD* pancreatoduodenectomy.

### Clinical outcomes

Clinical outcomes are shown in Table 5. The complete resection rate was significantly lower for the EP group than for the PD group (48.8% vs. 100%; p < 0.001). Although not significant, patients in the EP group tended to have a higher recurrence rate than those in the PD group (16.3% vs. 3.2%; p = 0.128). In both groups, all tumor recurrences were diagnosed as local recurrence. Of the 7 cases of recurrence in the EP group, one had adenocarcinoma and six had adenomas as the final histopathological result after EP. Recurrence in the EP group was diagnosed by endoscopic biopsy, and all recurrent tissues were adenoma. Recurrence after EP was treated with APC in six patients and by additional EP in one patient. Among them, one patient experienced recurrence 2 years after APC treatment, and underwent APC again; eight years have passed since the additional treatment, and no recurrence has been observed. Detailed information on the EP group is shown in the Supplementary Table S1. In the PD group, 1 T1a adenocarcinoma patient (limited to the sphincter of Oddi beyond the mucosa of the Ampulla of Vater) was diagnosed with local recurrence on CT 15 months after surgery and was treated with chemotherapy. However, the patient discontinued treatment because of tumor progression and died 28 months after surgery.

**Table 5:** Clinical outcomes.

	ALL n = 74	EP n = 43	PD n = 31	p-value
Complete resection, n (%)	55 (74.3)	21 (48.8)	31 (100)	< 0.001
Recurrence, n (%)	8 (10.8)	7 (16.3)	1 (3.2)	0.128
Median duration of recurrence, month (range)	6.2 (1.0–26.1)	6.1 (1.0–26.1)	15.9 (15.9)	–
**Additional treatment for recurrence, n (%)**				
APC	6 (8.1)	6 (13.9)	–	
EP	1 (1.4)	1 (2.3)	–	
Chemotherapy	1 (1.4)	–	1 (3.2)	
Median length of hospital stay, day (range)	17 (7–68)	11 (7–57)	42 (14–68)	< 0.001
Readmission, n (%)	19 (25.7)	12 (27.9)	7 (22.6)	0.788
Death, n (%)	4 (5.4)	1 (2.3)	3 (9.7)	0.302
Local recurrence	1 (1.4)	–	1 (3.2)	
**Pancreatic cancer**	1 (1.4)	–	1 (3.2)	
Heart failure	1 (1.4)	1 (2.3)	–	
Herpes encephalitis	1 (1.4)	–	1 (3.2)	
Median observation period, month (range)	52.0 (6.5–157.6)	38.8 (6.5–157.6)	59.8 (9.3–143.6)	0.123

*APC* algon plasma coagulation, *EP* endoscopic papillectomy, *PD* pancreatoduodenectomy.

The median length of hospital stay was significantly shorter in the EP group than in the PD group (11 days vs. 42 days; p < 0.001). There was no significant difference in the number of readmissions between the EP and PD groups (27.9% vs. 22.6%; p = 0.788).

**Figure 2:**
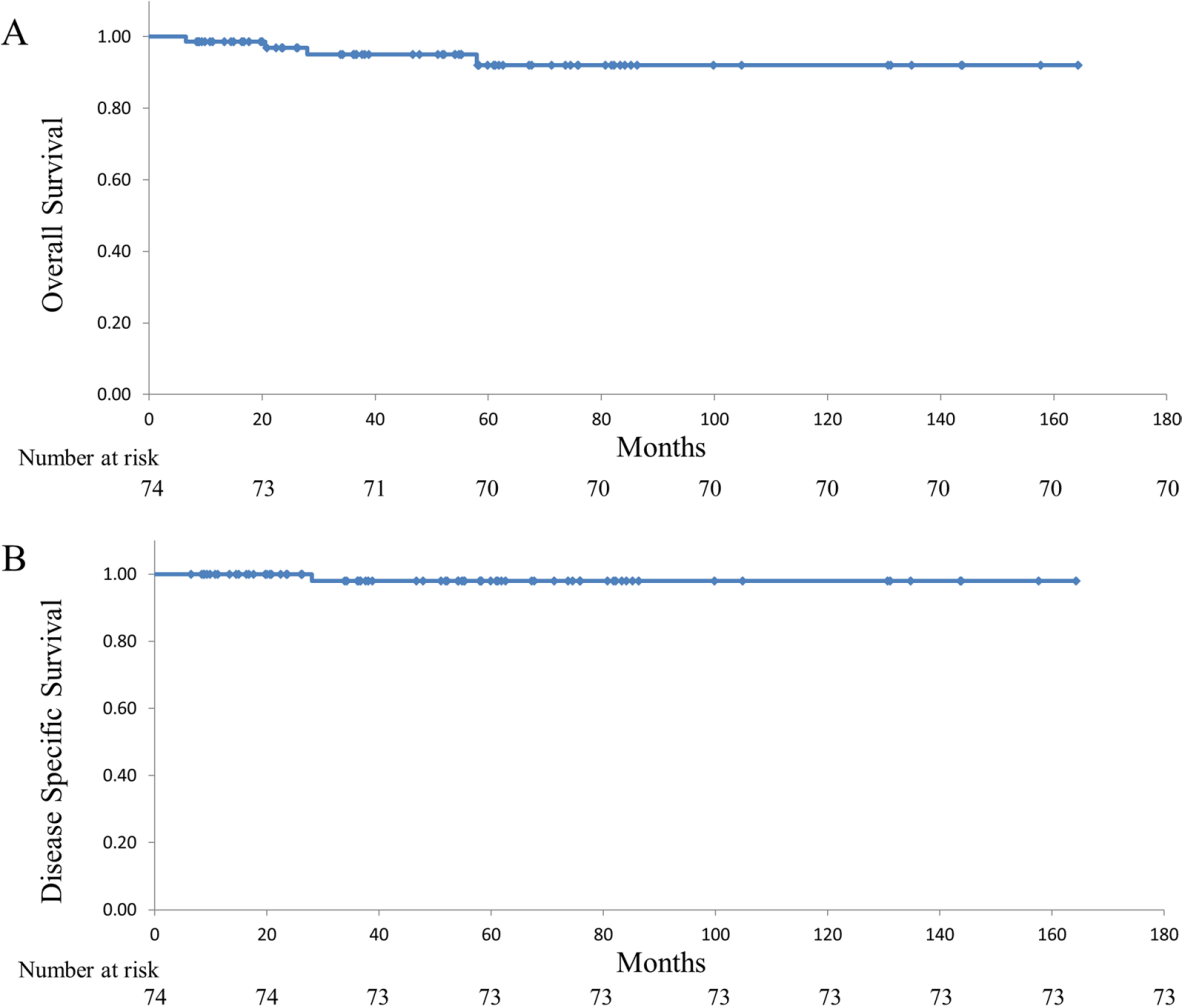
Estimated overall survival (**A**) and disease specific survival (**B**) of patients with an early ampullary tumor.

Four (5.4%, 4/74) patients died during the observation period. There were three deaths in the PD group; only one was an ampullary tumor-related death. The remaining two cases were due to pancreatic cancer and herpes encephalitis. In the EP group, one death due to heart failure was noted. The estimated five-year OS and DSS were 92% (Fig. 2A) and 98% (Fig. 2B), respectively. Because the rate of the adenocarcinoma in the EP and PD groups were different, the survival rates between the two groups were not compared. There was no significant difference in the observation period between the EP and PD groups (38.8 mo vs 59.8 mo; p = 0.123).

### Adverse events

Post-procedural adverse events are summarized in Table 6. There were no adverse events related to death. With regards to early adverse events in the EP group, pancreatitis was seen in 13 patients (30.2%), of which one patient (2.3%) was diagnosed severe pancreatitis. In addition, postoperative hemorrhage was observed in 10 patients (23.3%), and perforation was observed in one patient (2.3%). In patients with PD, postoperative hemorrhage was observed in two patients (6.5%), pancreatic fistula in 13 patients (41.9%), and bile leak in one patient (3.2%). All pancreatic fistulas were grade B. Comparing the early adverse events of EP and PD, although CDC grade I adverse events were significantly lower in EP (2.3% vs. 64.2%; p < 0.001), there was no significant difference in the severity of adverse events between the two groups (2.3% vs. 10.7%; p = 0.302). The CCI was significantly lower in the EP group than in the PD group (14.8 vs 22.6%; p = 0.002). Late adverse events of EP included cholangitis in 2 cases (4.7%) and pancreatitis in 4 cases (9.3%) cases. The cause of cholangitis was papillary stenosis in both cases. The cause of pancreatitis was endoscopic biopsy in 3 cases and papillary stenosis in 1 case. In the PD group, three (9.7%) cases of cholangitis and one (3.2%) case of pancreatitis were observed. In addition, one case of perforation of an anastomotic ulcer requiring emergency surgery was observed 6 months after PD.

**Table 6:** Post-procedural complications.

	ALL n = 74	EP n = 43	PD n = 31	p-value
**Early adverse events, n (%)**	–	–	–	–
Adverse events of EP	–	–	–	–
**Pancreatitis**	–	13 (30.2)	–	–
Mild/moderate	–	12 (27.9)	–	–
Severe	–	1 (2.3)	–	–
Hemorrhage		10 (23.2)		
Perforation	–	1 (2.3)	–	–
**Adverse events of PD**	–	–		–
Hemorrhage	–	–	2 (6.5)	–
Pancreatic fistula	–	–	13 (41.9)	–
Bile leak	–	–	1 (3.2)	–
**CDC**	–	–	–	–
I	19 (25.6)	1 (2.3)	18 (58.0)	< 0.001
II	27 (36.5)	14 (32.6)	13 (41.9)	0.318
IIIa	17 (23.0)	9 (20.9)	8 (25.8)	0.571
IIIb	1 (1.4)	0	1 (3.2)	0.152
IVa	3 (4.1)	1 (2.3)	2 (6.5)	0.557
IVb, V	0	0	0	–
Severe complications	4 (5.4)	1 (2.3)	3 (9.6)	0.302
Median CCI (range)	20.9 (0–54.1)	14.8 (0–54.1)	22.6 (0–43.3)	0.002
**Late adverse events n (%)**	–	–	–	–
Cholangitis	–	2 (4.7)	3 (9.6)	–
Pancreatitis	–	4 (9.3)	1 (3.2)	–
Perforation	–	–	1 (3.2)	–

*CDC* Clavien–Dindo classification, *CCI* comprehensive complication index, *EP* endoscopic papillectomy, *PD* pancreatoduodenectomy.

## Discussion

Herein, we investigated the clinical outcomes of patients who underwent EP or PD for early ampullary tumors to clarify the advantages of EP for early ampullary tumors. Patients who underwent EP or PD for early ampullary tumors had favorable prognoses, with estimated 5-year OS and DSS rates of 92% and 98%, respectively. In the EP group, the complete resection rate was lower and the recurrence rate was higher, but recurrent tumors were controllable by endoscopic treatment. Although there was no significant difference between EP and PD for severe adverse events, EP patients had fewer complications and shorter hospital stay post treatment than PD patients.

Yoon et al. reported that among patients who underwent EP or PD for T1 or lower ampullary cancer, T1 cancer showed 10.7% lymph node metastases, but those who received EP had no cancer recurrence or disease-related death^21^. Hwang et al. compared patients who received EP alone for ampullary carcinoma (Tis and T1a) with those who received additional PD, and reported that the 5-year disease-free and cancer-free survival rates were 79.1% vs. 87.4% (p = 0.111) and 93.5% vs. 87.4% (p = 0.726), respectively^22^. Dubois et al. compared the postoperative adverse events of EP and surgical ampullectomy using the CDC and CCI, and reported a significantly lower adverse events rate with EP^23^. However, although EP is considered to be a less invasive treatment than PD, there are no reports comparing EP and PD for ampullary tumors to clarify the advantages of EP. In this study, we compared EP and PD adverse events using a common scale and investigated clinical outcomes, and found that the EP group had fewer complications and shorter hospital stays.

The current study showed that the complete resection rate was significantly lower in patients with EP. There was no significant difference in the recurrence rate, but it tended to be higher in the EP group. Kawashima et al.^24^ reported that the recurrence rate of patients who underwent EP for ampullary tumors, including early carcinoma, was 16.9% after 5 years. The burning effect of EP was considered to be the reason why the complete resection rate of EP cases was low at this study. The post-EP resection margins of ampullary tumors are often positive or uncertain because of the burning effect of EP. However, in a previous study, we reported that all recurrent ampullary tumors after EP, including the resected margin positive or uncertain cases, were successfully treated with APC, and there was no local or lymph node recurrence after APC^25^. In this study, seven patients with recurrence after EP were successfully treated with APC or additional EP. Although excluded from this study, five patients underwent additional PD because the resected margin after EP was uncertain or positive. Four of those cases had no residual tumor in the post-PD specimens. As a result, we propose that early ampullary tumors that cannot be completely resected by EP can be managed by endoscopic treatment rather than by additional PD. However, long-term careful follow-up is required because of the possibility of recurrence after EP.

T1a ampullary carcinoma is difficult to be cured by EP due to the presence of lymph node metastasis with a certain probability, and is still considered to be an indication for PD. Yamamoto et al. reported that EP is reliable for the curative treatment of T1a ampullary carcinoma (limited to ampulla of Vater), but there is no preoperative modality that can definitively diagnose whether or not the tumor has invaded the sphincter of Oddi^12^. Moreover, there are few reports on the long-term prognosis of ampullary carcinoma after EP, because additional PD is often performed in cases of ampullary carcinoma diagnosed after EP. In the present study, none of the 11 T1a ampullary carcinoma in which EP was performed had invasion of the sphincter of Oddi. All of these cases had a good clinical course, and the one case of recurrence was controlled by endoscopic treatment. Of the 21 T1a cases of PD, 11 had sphincter of Oddi invasion. One of them showed lymphovascular invasion and died of primary disease. These results suggest that even if the final pathological result after EP is T1a, additional PD may not be necessary if there is no invasion of the sphincter of Oddi. In order to standardize the use of EP for ampullary carcinoma, it is desirable to develop a method that can accurately diagnose invasion of the cancer to the sphincter of Oddi.

The present study had some limitations. First, this was a single-center retrospective study. Second, due to the small sample size of this study, the results could not be generalized. Third, since the CDC and CCI index were originally designed to assess surgical complications, the validity of their used in this study to compare endoscopic and surgical complications was unclear. Fourth, there was a large difference in the number of ampullary carcinoma cases between the EP and PD groups. Due to these selection biases, the survival rate could not be statistically examined. To investigate this further, a multicenter study of patients who underwent EP for early ampullary carcinoma or were diagnosed with early ampullary carcinoma after EP and underwent additional PD is needed.

In conclusion, patients who underwent EP or PD for early ampullary tumors had a favorable prognosis. Furthermore, the EP group had fewer complications and a significantly shorter hospital stay than the PD group. This suggests that EP is a less invasive treatment and a useful option compared to PD for the treatment of ampullary tumors in select cases.

## Supplementary Information

Supplementary Table S1.

## Data Availability

The datasets used and/or analyzed during the current study are available from the corresponding author on reasonable request.
