# Supplementary material for: Advantage of endoscopic papillectomy for ampullary tumors as an alternative treatment for pancreatoduodenectomy
Source: Sci Rep. 2022 Sep 7;12:15134. doi: 10.1038/s41598-022-19439-3 (PMC9452518; doi:10.1038/s41598-022-19439-3)
Supplement: Supplementary file 1 — Supplementary Table S1. [file 41598_2022_19439_MOESM1_ESM.docx]

**Supplementary table S1. The background date for patients who underwent EP**

|  | n=43 |
| --- | --- |
| Type of resection, n(%) |  |
| en bloc | 40 (93.0) |
| piecemeal | 3 (7.0) |
| Stent placemant, n(%) |  |
| pancreatic stent | 8 (18.6) |
| billiary stent | 3 (7.0) |
| double stent | 31 (72.1) |
| none | 1(2.3) |
| Preoperative biopsy, n |  |
| adenoma/borderline/adenocarcinoma | 30/9/4 |
| Final pathological diagnosis, n |  |
| adenoma/adenocarcinoma | 32/11 |
| Pathological evaluation of resected margin, n |  |
| positive/uncertain/negative | 10/12/21 |

Abbreviations: EP, endoscopic papillectomy
